# Radiological and Pathological Features of Cyst Formation in Idiopathic Multicentric Castleman Disease

**DOI:** 10.3390/arm91020014

**Published:** 2023-04-19

**Authors:** Ryota Otoshi, Akimasa Sekine, Tatsuya Muraoka, Tae Iwasawa, Tamiko Takemura, Shoichiro Matsushita, Koji Okudela, Hideya Kitamura, Tomohisa Baba, Takashi Ogura

**Affiliations:** 1 Department of Respiratory Medicine, Kanagawa Cardiovascular and Respiratory Center, Yokohama 236-0051, Japan; 2 Department of Radiology, Kanagawa Cardiovascular and Respiratory Center, Yokohama 236-0051, Japan; 3 Department of Pathology, Kanagawa Cardiovascular and Respiratory Center, Yokohama 236-0051, Japan; 4 Department of Radiology, St. Marianna University School of Medicine, Kawasaki 216-8511, Japan; 5 Department of Pathology, Yokohama City University Graduate School of Medicine, Yokohama 236-0004, Japan

**Keywords:** cyst formation, elastin, elastolysis, multicentric Castleman disease, plasma cell infiltration

## Abstract

**Highlights:**

**What are the main findings?**

- Pulmonary cysts in idiopathic Castleman disease (MCD) emerged from the area of ground-glass attenuation (GGA) on HRCT, and the cysts did not regress by treatment.
- The pathological evaluation showed a high degree of plasma cell infiltration and loss of elastic fibers around the cyst wall.

**What are the implications of the main findings?**

- The loss of elastic fibers may be an important factor in cyst formation in idiopathic MCD.
- Introducing anti-inflammatory therapy, such as tocilizumab, before pathologic loss of elastic fibers occurs might prevent irreversible cyst formation in MCD.

**Abstract:**

Introduction: Idiopathic multicentric Castleman disease (MCD) has been reported to form lung cysts at a relatively high rate. However, the radiological and pathological features of cystic formation in MCD are unclear. Methods: To clarify these questions, we retrospectively investigated the radiological and pathological findings of cysts in MCD patients. Eight consecutive patients who underwent surgical lung biopsies in our center from 2000 to 2019 were included. Results: The median age was 44.5 years, with three males and five females. On the initial computed tomography, cyst formation was found in seven patients (87.5%). All of the cysts were multiple, round, and thin walled, accompanying ground-glass attenuation (GGA) around cysts. In six patients (75%), cysts increased during their clinical courses, and the new cysts had emerged from GGA, although GGA was improved by treatment. In all four cases, whose pulmonary cysts could be pathologically evaluated, a marked plasma cell infiltration around the cyst wall, and loss of elastic fibers of the alveolar wall were observed. Conclusions: Pulmonary cysts emerged in the area of GGA pathologically consistent with plasma cell infiltration. Cysts in MCD may be formed by the loss of elastic fibers due to marked plasma cell infiltration and may be considered irreversible changes.

## 1. Introduction

Multicentric Castleman disease (MCD) is a rare polyclonal lymphoproliferative disease of unknown etiology, which affects multiple organs due to the overproduction of interleukin-6 (IL-6) [1,2,3,4,5]. MCD is divided into two subtypes: human herpesvirus-8-associated MCD and idiopathic MCD [6,7]. There are only a few reports of radiological features of diffuse lung disease in idiopathic MCD because of its rare prevalence [8,9,10]. The limited studies reported that the interlobular septal thickening and multiple centrilobular nodules were present in most cases of idiopathic MCD [11,12]. Similarly, thin-walled cysts are also reported to be found at relatively high rates (59–83%). In addition, there are two case reports of idiopathic MCD with diffuse lung cysts [13,14]. Thus, thin-walled cysts are considered to be one of the characteristic findings in idiopathic MCD. However, the radiological and pathological features of cystic formation in MCD are unclear.

In the present study, to clarify these questions, we retrospectively investigated the radiological and pathological features of idiopathic MCD in our hospital.

## 2. Materials and Methods

### 2.1. Patients

This is a retrospective study of eight patients with idiopathic MCD. We retrospectively reviewed all consecutive patients who received a definitive diagnosis of idiopathic MCD by surgical lymph node biopsy and, simultaneously, underwent surgical lung biopsy (SLB) in our center from January 2000 to December 2019. The following information was collected from the patient’s medical records: age, sex, smoking history, symptoms at first visit, duration from onset to first visit, physical examination, serological data, radiological findings, and pathological findings of surgical lung biopsy. For objective assessment, the diagnosis of idiopathic MCD was made according to the international diagnostic criteria by Fajgenbaum et al. [3].

### 2.2. Radiological Findings

High-resolution computed tomography (HRCT) findings at the initial visit were evaluated by chest radiologists. Two radiologists (T.I. and S.M.) assessed the HRCT findings independently, and in case of disagreement, the final decision was made through consultation. HRCT scans were performed for each patient during breath hold at full inspiration; 1.0 mm thick sections were collected throughout the lungs. With reference to previous reports, the presence of mediastinal lymphadenopathy, interlobular septal thickening, centrilobular nodules, bronchovascular bundles thickening, cysts, ground-glass attenuation (GGA), consolidation, bronchiectasis, air trapping, and pleural effusion were evaluated [11,12]. The cysts were also evaluated for detailed information, such as location, number, size, and surrounding appearance. In addition, these findings were also evaluated for changes over time.

### 2.3. Pathological Findings

Two lung pathologists (T.T. and K.O.) reviewed the histological sections of surgical lung biopsy specimens independently and discussed among themselves to reach one final pathological interpretation for each case. Histological sections of surgical lung biopsy specimens were stained with hematoxylin-eosin and elastic van Gieson. The following pathological features were semiquantitatively graded as 0 (absent), 1 (mild), 2 (moderate), or 3 (severe): inflammation in interstitial area (bronchovascular bundle, interlobular septa, and alveolar wall), interstitial fibrosis, and inflammatory cell infiltration (plasma cell, lymphoid follicles with germinal center, and eosinophils). The extent of inflammatory cell infiltration of each lesion was evaluated in three randomly selected fields and graded based on the following numbers; (i) plasma cell [grade 0, 0/high-power field (HPF); grade 1, 1–19/HPF; grade 2, 20–39/HPF; grade 3, 40-/HPF], (ii) lymphoid follicles with germinal center(grade 0, 0/HPF; grade 1, 1–2/HPF; grade 2, 3–5/HPF; grade 3, 6-/HPF), and (iii) eosinophils (grade 0, 0/HPF; grade 1, 1–4/HPF; grade 2, 5–9/HPF; grade 3, 10-/HPF). In addition, cases in which the cyst was pathologically evaluable were investigated for detailed information, such as cell infiltration around the cyst, structural changes of the alveolar wall, and association with bronchioles.

### 2.4. Statistical Analysis

Categorical data are presented as numbers (percentages), and continuous data are presented as medians, in the text and figures.

## 3. Results

### 3.1. Baseline Characteristics

The baseline characteristics of the patients are shown in Table 1. There were eight patients who were diagnosed with idiopathic MCD by lymph node biopsy and, at the same time, underwent surgical lung biopsy. The median age was 44.5 years; there were three males and five females. There were three current or former smokers and five were never smokers. Of the eight patients, seven (87.5%) had symptoms, such as cough, dyspnea, and fatigue. The blood tests revealed elevated C-reactive protein levels, immunoglobulin G, IL-6, and hypoalbuminemia in all patients, and the immunoelectrophoretic analysis confirmed polyclonal hypergammaglobulinemia. Of the eight patients, six (75%) had anemia, five (62.5%) had thrombocytosis or thrombopenia, one (12.5%) had renal dysfunction, and five (62.5%) had elevated KL-6.

### 3.2. Radiological Findings on the Initial Visit

The summary of computed tomography (CT) findings of the eight patients at the first visit is shown in Table 2. Mediastinal lymphadenopathy, interlobular septal thickening, and centrilobular nodules were found in all patients. Cyst formation was observed in seven patients (87.5%). During the initial examination, only six patients underwent expiratory CT, and five of them (83.3%) had air trapping.

**Figure 1:**
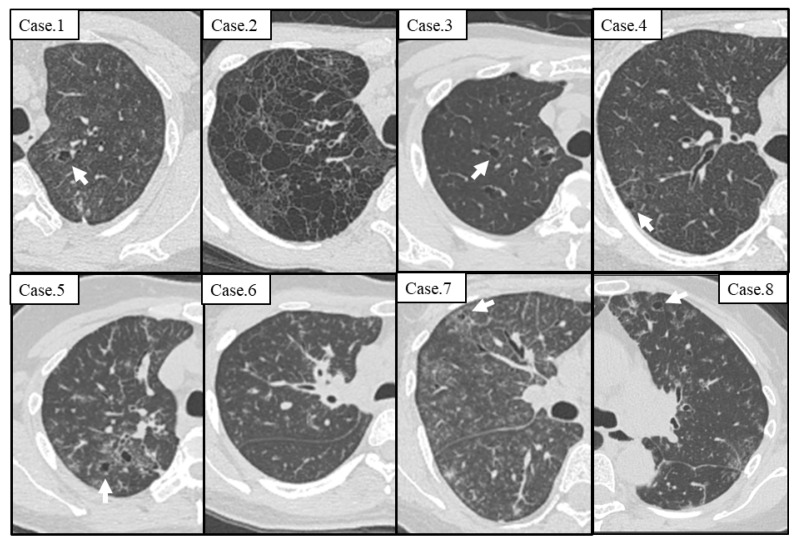
Cysts on Computed tomography at the first visit in 8 cases. Seven cases (87.5%) other than Case 6 had thin-walled cysts (arrow). There was ground-glass attenuation around cysts.

The initial chest CT of all patients are shown in Figure 1. Seven cases (87.5%) had thin-walled cysts at the first visit. In addition, a case without a cyst on the initial CT (Case 6) also developed a cyst thereafter. Table 3 presents the detailed characteristics of the cyst on chest CT. In all cases, the cysts were multiple and thin walled, most of which were less than 10 mm in diameter, and there were GGA around the cysts. Cysts were frequently distributed in the upper lobes (7/7, 100%) and along the interstitial area, including bronchovascular bundles and veins (6/7, 85.7%). In one patient with numerous cysts (Case 2), the cysts varied in size and shape and were randomly distributed.

### 3.3. Radiological Course of Cysts

**Figure 2:**
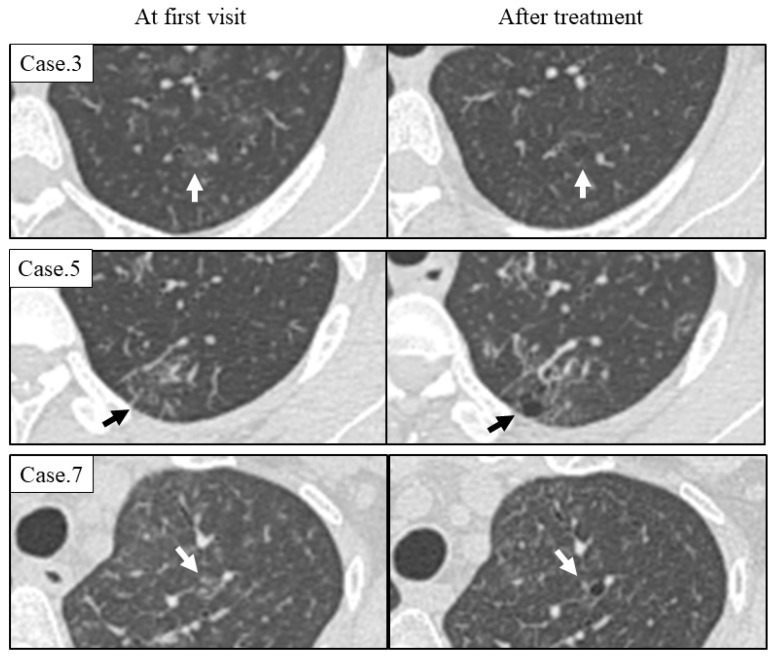
Chest high-resolution computed tomography imaging of cases with newly emerged cysts during the course. A new cyst emerged from the site of ground-glass attenuation on HRCT at the time of initial diagnosis (arrow). The cyst appeared despite the improvement of the ground-glass attenuation with treatment.

Seven patients were treated with anti-inflammatory therapy, including prednisolone and tocilizumab, and their GGA was improved. However, in six patients (75%), the number of cysts increased during the course (Table 3), and new cysts emerged from the area of GGA as shown in Figure 2.

### 3.4. Pathological Findings

Table 4 shows the summary of pathological findings of lung biopsy in the eight patients. In all patients, the pathological pattern of MCD was plasma cell type, and all cases had prominent plasma cell infiltration in the interstitial area. Although the lesions tended to be severe in the peribronchovascular bundle, some cases had advanced lesions in other interstitial areas, such as interlobular septa and alveolar wall. Fibrosis was relatively mild in most cases.

**Figure 3:**
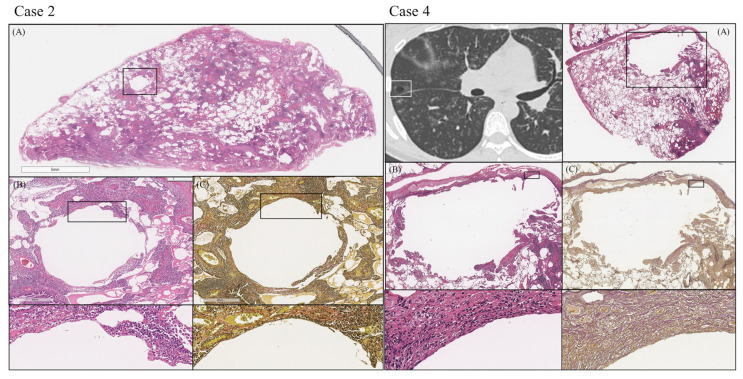
Pathological images of lung cyst. (Case 2 and Case 4). Case 2 is a case of multiple cysts and Case 4 is a case of biopsy targeting a cystic lesion. Plasma cell infiltration and multiple lymphoid follicles with germinal center are present mainly in the interstitial area (**A**). Hematoxylin-eosin staining showed a high degree of plasma cell infiltration around the cyst (**B**), and elastic-van Gieson staining showed a loss of elastic fibers in the alveolar wall (**C**).

Of the eight patients, there were four cases whose pulmonary cysts could be pathologically evaluated. In all four cases, a remarkably high degree of plasma cell infiltration around the cyst wall, loss of elastic fibers of the alveolar wall, and the opening of bronchioles to the cyst were observed (Table 4). Figure 3 displays the pathological images of the two cases (Case two and Case four) as examples.

#### Case Presentation (Case 8)

**Figure 4:**
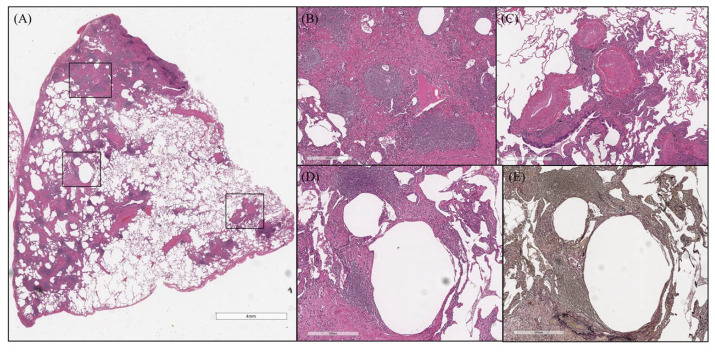
Surgical lung biopsy of case presentation (Case 8). Inflammatory cell infiltration and lymphoid follicles with germinal center presented mainly in the interstitial area, such as bronchovascular bundle and interlobular septa (**A**–**C**). There was a remarkably high degree of plasma cell infiltration around the cyst wall and loss of elastic fibers of the alveolar wall (**D**,**E**).

Here, a typical case of idiopathic MCD with increased cysts during the course is presented. In August 2017, a 57-year old nonsmoking woman visited our hospital with a complaint of fatigue and dyspnea on exertion. HRCT showed a wide range of GGA, interlobular septal thickening, bronchovascular bundle thickening, and multiple centrilobular nodules (Figure 1). Thin-walled cysts were also found sporadically, and there was GGA around the cysts. Surgical lung biopsy revealed inflammatory cell infiltration and lymphoid follicles with germinal center mainly in the interstitial area, such as bronchovascular bundle and interlobular septa (Figure 4A–C). A remarkably high degree of plasma cell infiltration around the cyst wall and loss of elastic fibers of the alveolar wall were observed (Figure 4D,E).

**Figure 5:**
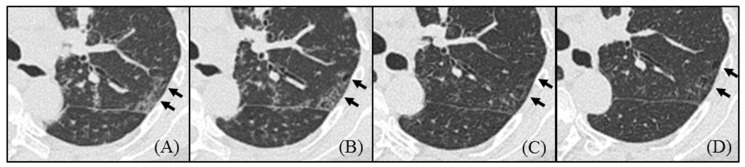
The course of high-resolution computed tomography imaging of case presentation (Case 8). Initial CT showed ground-glass attenuation (**A**), which regressed after treatment with Tocilizumab (**B**–**D**). However, new cysts gradually emerged fromGGA regressed with treatment (**B**–**D**).

Treatment with tocilizumab was started in March 2018, which led to an improvement in her symptoms, respiratory function, and inflammatory parameters. Improvement of GGA, interlobular septal thickening, and centrilobular nodules was also shown in HRCT. However, the cysts continued to increase after MCD treatment. Of note, a cyst gradually emerged from GGA that regressed with treatment (Figure 5).

## 4. Discussion

In the present study, we evaluated the radiological and pathological features of cystic formation in idiopathic MCD. In our study, three clinically important findings were identified.

First, there was GGA around the cysts of idiopathic MCD, and more importantly, the new cysts had emerged from the area of GGA. Second, the pathological evaluation showed a high degree of plasma cell infiltration consistent with radiologically GGA and loss of elastic fibers of the alveolar wall around the cyst wall. Finally, the cysts did not regress by treatment although other lesions, such as GGA, centrilobular nodules, and interlobular septal thickening, were improved.

The limited studies reported that GGA and cysts were found in most cases of idiopathic MCD on CT, but there are no reports on the relationship between GGA and cysts [11,12]. In the present study, cysts emerged from the areas of the GGA. The mechanism of cyst formation in MCD is unclear, but our study suggests a close association between the progression of GGA and the formation of cysts. Additionally, in cases whose pulmonary cysts could be pathologically evaluated, loss of elastic fibers in the alveolar wall was found, while in cases where no cysts were obtained by SLB, the elastic fibers were preserved. Thus, the loss of elastic fibers may also be an important factor in cyst formation in MCD. In other cystic diseases, including light chain deposition disease, Langerhans’ cell histiocytosis, and lymphangioleiomyomatosis, pathological loss of elastic fibers caused by the proteolytic action of metalloproteinases expressed by inflammatory cells has been reported as a mechanism of cyst formation [15,16,17]. Although the mechanisms of the loss of elastic fibers in idiopathic MCD are unclear, as in the other cystic diseases mentioned above, an unknown proteolytic enzyme might degrade the elastic fibers, resulting in cyst formation.

In the present study, the cysts had not regressed in all patients with idiopathic MCD. Rather, in the seven cases treated, cysts increased in five cases, despite improvement in the other lesions. This fact suggests that the cystic lesion of MCD is an irreversible change. As noted above, the cysts may not regress once the elastic fibers have disappeared, suggesting the importance of early therapeutic intervention for MCD. Introducing anti-inflammatory therapy, such as tocilizumab, before pathologic loss of elastic fibers occurs might prevent irreversible cyst formation in MCD.

This study has several limitations. First, this study was a small retrospective study at a single institution, and our results may have been influenced because of inadequate power. Second, cysts were not pathologically evaluated in all patients because the cyst was unintentionally obtained by SLB. Therefore, large-scale studies are required to confirm our results.

## 5. Conclusions

We evaluated the radiological and pathological features of idiopathic MCD. In idiopathic MCD, pulmonary cysts emerged in the area of GGA pathologically consistent with prominent plasma cell infiltration and were considered to be irreversible changes. The loss of elastic fibers in the alveolar wall may lead to cyst formation. Hence, further large-scale study is needed to establish the results of this study.

## Figures and Tables

**Table 1:** Summary of Patient Characteristics of 8 cases.

Case	1	2	3	4	5	6	7	8	Median
**Age**	33	40	48	36	44	45	48	57	44.5
**Sex**	Male	Male	Male	Female	Female	Female	Female	Female	
**Smoking history**	Never	Never	Current	Never	Never	Former	Former	Never	
Pack years	0	0	28	0	0	1	22	0	
**Symptom**	Cough	Dyspnea	Chest pain	Fatigue	Nothing	Cough	Fatigue	Dyspnea	
**Duration from onset to first visit (months)**	45	274	2	9	15	8	62	60	30
**Laboratory data**									
Hemoglobin (g/dL)	15.4	8.0	11.8	10.4	10.6	10.8	11.8	8.9	10.7
Platelet (×10^4^/uL)	24.3	42.6	53.6	42.8	28.0	49.6	39.5	47.8	42.7
CRP (mg/dL)	4.6	7.9	6.3	7.4	6.2	1.5	3.9	7.4	6.3
Creatinine (mg/dL)	0.89	1.38	0.68	0.51	0.55	0.65	0.69	0.50	0.67
Total protein	10.1	11.9	10.3	10.5	9.3	9.1	12.0	8.9	10.2
Albumin (g/dL)	3.0	1.7	2.7	2.6	2.8	3.0	2.9	2.5	2.8
IgG (mg/dL)	4341	8184	4779	5169	4730	3988	5974	3846	4755
IgG4 (mg/dL)	57	1812	416	242	N.E.	94	72	142	142
KL-6 (U/mL)	1453	973	285	574	256	448	507	1172	540.5
IL-6 (pg/mL)	16	20	28	17	16	10	14	35	16.5
Anti-nuclear antibodies	0	160	0	40	0	40	40	80	
Rheumatoid factor (IU/mL)	23	17	13	7	11	9	14	23	
Specific autoantibody	–	RNP	–	RNP, SS-A	–	–	–	–	
**Diagnosis of autoimmune disease**	–	–	–	–	–	–	–	–	
**Diagnosis of malignant disease**	–	–	–	–	–	–	–	–	
**Infectious disease**									
HIV antigen	–	–	–	–	–	–	–	–	
HHV-8 qPCR	–	–	–	–	N.E.		–	–	

Abbreviations: CRP, C-reactive protein; HHV-8, human herpesvirus 8; HIV, human immunodeficiency virus; IgG, immunoglobulin G; IL, interleukin; N.E., not evaluable.

**Table 2:** Summary of CT findings at the first visit.

CT Findings	*n* = 8
Mediastinal lymphadenopathy	8 (100%)
Interlobular septal thickening	8 (100%)
Centrilobular nodules	8 (100%)
Ground-grass attenuation	8 (100%)
Cyst formation	7 (87.5%)
Thickening of the bronchovascular bundles	6 (75%)
Air trapping (*n* = 6)	5 (83.3%)
Consolidation	1 (12.5%)
Bronchiectasis	1 (12.5%)
Pleural effusion	0 (0%)

Abbreviations: CT, computed tomography. Categorical data are presented as numbers (percentages).

**Table 3:** Summary of characteristics of cysts.

Case	Detail Characteristics of Cyst at First Visit							Clinical Course	
	Cyst	Number	Size	Wall	GGA around Cysts	Distribution		Treatment	The Number of Cysts
						Upper /Lower	Subpleural Area /Interstitial Area /Random		
1	+	2	8 mm	<2 mm	+	Upper	Interstitial area	TCZ	No change
2	+	>100	3–30 mm	<2 mm	+	Upper	Random	TCZ	No change
3	+	25	2–10 mm	<2 mm	+	Upper	Interstitial area	TCZ	Increase
4	+	12	2–8 mm	<2 mm	+	Upper	Interstitial area	PSL + TCZ	Increase
5	+	14	3–8 mm	<2 mm	+	Upper	Interstitial area	Observation	Increase
6	−	NE	NE	NE	NE	NE	NE	TCZ	Increase
7	+	3	2–3 mm	<2 mm	+	Upper	Interstitial area	TCZ	Increase
8	+	32	3–9 mm	<2 mm	+	Upper	Interstitial area	TCZ	Increase
Summary	7/8 (87.5%)	1–9; 2 10–99; 4 >100; 1	<10 mm; 6 >10 mm; 1	<2 mm; 7 >2 mm; 0	7/7 (100%)	Upper; 7 Lower; 0	Subpleural; 0 Interstitial; 6 Random; 1	TCZ; 6 PSL + TCZ; 1 Observation; 1	Increase; 6 No change; 2 Decrease; 0

Abbreviations: GGO, ground-glass attenuation; PSL, prednisolone; TCZ, tocilizumab; NE, not evaluated. Data are presented as number of patients (Patients).

**Table 4:** Summary of the Pathological findings.

Pathological Findings	*n* = 8
**Type (Hypervascular/ Mixed/ Plasmacytic)**	0/0/8
**Distribution**	
Bronchovascular bundle (Grade 0/1/2/3)	0/0/7/1
Interlobular septa (Grade 0/1/2/3)	0/3/3/2
Alveolar wall (Grade 0/1/2/3)	0/5/0/3
**Fibrosis (Grade 0/1/2/3)**	1/5/2/0
**Cell infiltration**	
Plasma cell (Grade 0/1/2/3)	0/0/0/8
Lymphoid follicles with germinal center (Grade 0/1/2/3)	0/4/2/2
Eosinophils (Grade 0/1/2/3)	3/4/1/0
**Characteristics of cyst wall**	***n* = 4**
Prominent infiltration of plasma cells	4 (100%)
Prominent infiltration of macrophages	0 (0%)
Granuloma	0 (0%)
Opening of bronchi	4 (100%)
Loss of elastic fibers	4 (100%)

Data are presented as numbers of patients (percentages). The higher grade means more severe change in pathological assessment.

## Data Availability

The data presented in this study are available on request from the corresponding author.
